# Technical Feasibility of Acoustic Coordinated Reset Therapy for Tinnitus Delivered via Hearing Aids: A Case Study

**DOI:** 10.1155/2017/5304242

**Published:** 2017-03-30

**Authors:** Christian Hauptmann, Mark Williams, Federica Vinciati, Markus Haller

**Affiliations:** ^1^Institute of Neuroscience and Medicine-Neuromodulation (INM-7), Jülich Research Center, Jülich, Germany; ^2^DESYNCRA Technologies Ltd., London, UK; ^3^DESYNCRA Operating GmbH, Bad Neuenahr-Ahrweiler, Germany; ^4^The Tinnitus Clinic Ltd., London, UK

## Abstract

Primary tinnitus has a severe negative influence on the quality of life of a substantial portion of the general population. When acoustic coordinated reset (CR) neuromodulation stimuli are delivered for several hours per day over several weeks a clinically significant symptom reduction in patients with primary tonal tinnitus has been reported by several clinical sites. Here, we reported the first case where CR neuromodulation was delivered through a hearing aid. A 52-year-old man with chronic primary tonal tinnitus was previously considered untreatable with sound therapy. He initially received the classic CR treatment protocol with signals delivered with the separate proprietary device with his hearing aids removed during treatment. He was subsequently treated with the therapy being deployed through a set of contemporary hearing aids. After 5 months of classic CR treatment with the separate custom device, the THI and VAS_L/A_ scores worsened by 57% and 13%/14%, respectively. Using the hearing aid without CR treatment for 5 months no change in tinnitus symptoms was observed. However, after three months of CR treatment delivered through the hearing aids, the THI and VAS_L/A_ scores were reduced by 70% and 32%/32%, respectively.

## 1. Introduction

Primary (subjective) tinnitus is an idiopathic symptom that may or may not be associated with sensorineural hearing loss [1]. The prevalence of chronic primary tinnitus is 10% to 15% of the general population [2] with a range of severity. Approximately 2% of the general population experience a severely impaired quality of life due to this condition and seek professional help [1, 3]. Secondary (objective) tinnitus refers to tinnitus associated with an identifiable organic condition other than sensorineural hearing loss [1]. Current tinnitus definitions and interventions are specified in detail in a widely accepted consensus guideline [4]. Accordingly, the only interventions with evidence-based outcomes are cognitive behavioral therapy [5], hearing aids [6], and sound therapy that can include sound maskers [7].

Sensorineural hearing loss, especially that associated with noise exposure and aging, can result in a reduction in the number of functioning sensory hair cells in the cochlea. Although sensory hair cell loss can lead to a sensorineural hearing loss, it is highly unlikely that the hair cell loss itself is the source of the tinnitus. It is much more likely that the deafferentation process can trigger abnormal neuronal synchrony in the central nervous system and, if persistent, can lead to an abnormal upregulation of synaptic connectivity to other brain regions resulting in chronic primary tinnitus [8–10]. Chronic primary tinnitus is associated with altered spectral power of electroencephalography (EEG) or magnetoencephalography signals [9–16] observable over a large network of brain areas [17–20] but predominantly in the temporal cortex.

Acoustic coordinated reset (CR) therapy is a sound-based therapy supported by neuromodulation principals [21–23]. Utilizing the tonotopic organization of the auditory system, acoustic CR therapy uses sequences of acoustic tonal stimuli, namely, four different frequencies centered around the characteristic frequency of the patient's tinnitus percept, delivered in nonsimultaneous sequences several hours per day for several weeks [14]. The stimuli target areas of pathological neural synchrony within the central auditory system and are intended to induce a sustained reduction of the strength of tinnitus-related synaptic connectivity among neurons within the affected neuronal population [14, 19] after therapy completion. This form of therapy has been tested in several clinical studies [14, 24, 25]. These studies have shown that statistically and clinically significant improvements in tinnitus symptoms were obtained in a variety of outcome measures including visual analog scales (VAS) for tinnitus loudness (reduction of 53% during stimulation and 31% after stimulation), tinnitus annoyance (reduction of 49% during stimulation and 27% after stimulation), and tinnitus questionnaire results (reduction of 29% compared to baseline) [14, 15, 26]. The investigation of EEG activity has shown a significant decrease of abnormal EEG power spectra in the delta, theta, beta, and gamma bands to more normal levels [14, 16, 27], a statistically significant decrease of effective connectivity within frequency bands [19], and a statistically significant decrease of cross-frequency coupling [28]. In a large observational study of 200 patients at 23 clinical sites a statistically and clinically significant improvement in Tinnitus Impairment Questionnaire scores (a reduction by up to 38%) and in Clinical Global Improvement-Impression scales was observed (a reduction by up to 33%) [24].

In these studies, the daily therapy signals were delivered by a custom device (T30 CR neurostimulator) connected to earphones by cables, an arrangement that is not ideal. The use of the therapy devices requires that hearing aid patients remove their hearing aids during therapy so the benefits of the hearing aids are removed during the therapy, an important consideration because a large percentage of patients with chronic primary tinnitus also have a degree of hearing loss that warrants hearing aids and the use of hearing aids in tinnitus patients itself can reduce tinnitus symptoms [29–31]. Furthermore, few studies have shown that sound therapies delivered by hearing aids were successful in alleviating effects of tinnitus [32, 33].

We report results obtained by the delivery of acoustic CR therapy via hearing aids and results obtained by delivery via a separate proprietary sound system in a single hard to treat patient.

## 2. Case Report

**Figure 1:**
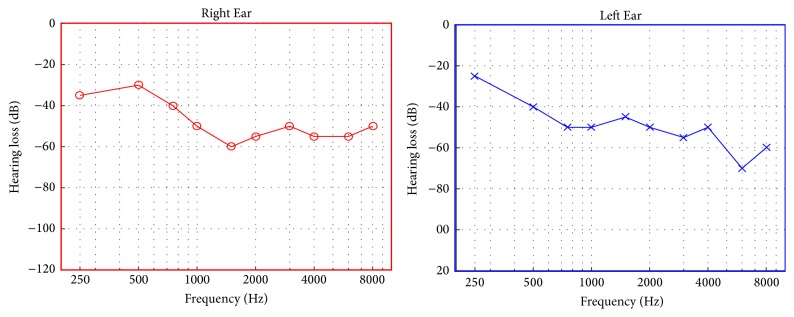
Audiograms of the patient obtained before starting the second treatment phase.

One male patient (52 years) with a diagnosis of idiopathic chronic primary bilateral tinnitus participated in this case study. His tinnitus symptoms started in 2005 at the age of 41 years. He presented bilateral, sensorineural, high frequency hearing loss with a pure-tone average of 49 dB HL in both ears (Figure 1).

### 2.1. Overview of Chronologic Sequence of Treatments

**Figure 2:**
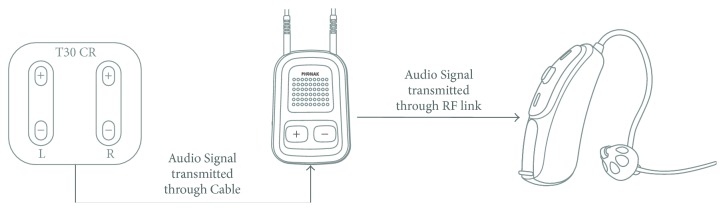
Device configuration. The tinnitus neuromodulation therapy device (neurostimulator T30 CR, left) applied the acoustic CR neuromodulation signals via cable to the “streamer” (Phonak ComPilot, middle) that in turn sent the therapy signals via radio frequency link to the two hearing aids (Phonak Audeo V50, right).

The patient wore hearing aids for 8 months prior to initial treatment with acoustic CR neuromodulation, relies on them for communication ability, and reported that they provide no benefits regarding tinnitus symptoms. The CR neuromodulation therapy was implemented for 19 weeks in 2012 with the standard acoustic stimuli delivered via a commercially available tinnitus device (T30 CR, ANM GmbH, Cologne, Germany). The tinnitus therapy required removal of his hearing aids during the therapy. Subsequently (2015-2016), he was treated for 12 weeks with the same neuromodulation therapy device (T30 CR, Neurotherapies Reset GmbH, Jülich, Germany, a company of the Brook Henderson Group Limited) but connected to an external “streamer” (Phonak ComPilot,) that was a component of commercially available hearing aids (Phonak Audeo V50 Sonova AG, Stäfa, Switzerland), Figure 2.

The streamer had a sampling rate of 22 kHz (stereo) and an audio bandwidth from 20 Hz to 10 kHz (Datasheet Phonak ComPilot), while the hearing aid (Phonak Audeo V50) had a bandwidth from 100 Hz to 9.2 kHz. These bandwidth limitations, well known for conventional hearing aid signal processing [34], may have additional limitations for the transfer of the CR therapy signals, especially in the high frequency range.

No further otolaryngology or audiology related issues were diagnosed while undergoing the two therapy phases and no pretreatments for tinnitus were reported except for the hearing aids that were fitted in July 2015. All treatments and hearing aid fittings were conducted by one of the authors (Mark Williams, Chief Audiologist at The Tinnitus Clinic, London, UK).

### 2.2. Acoustic CR Neuromodulation and Outcome Measures

The standard acoustic CR neuromodulation therapy signals were used in both therapy periods and consisted of four pure tones determined by the pitch matched tinnitus frequency. The frequencies of the four therapy tones, two below and two above the tinnitus frequency, were a percentage of the tinnitus frequency, namely, from 77% to 140%, presented sequentially in a randomized fashion at approximately 5 dB above the individual threshold at that frequency. The daily stimulation duration was 4–6 hours [14].

Tinnitus outcome measures used were the Tinnitus Handicap Inventory (THI) scores and the visual analog scale scores for tinnitus loudness (VAS_L_ on a scale from 0 to 100) and for tinnitus annoyance (VAS_A_ on a scale from 0 to 100) at baseline and after treatment. Please note that the sensitivity of the THI score has been challenged by Tyler et al. [35].

### 2.3. First Phase of Treatment with Acoustic CR Neuromodulation

**Figure 3:**
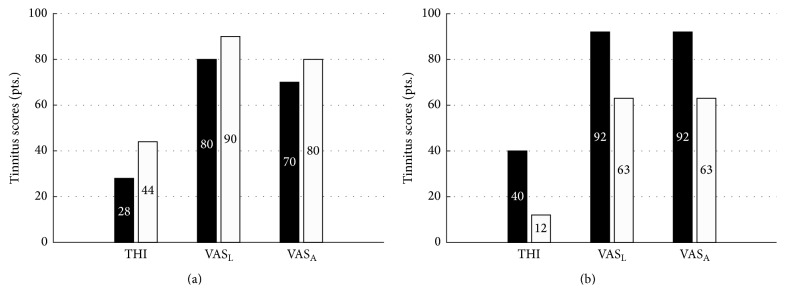
THI, VAS_L_, and VAS_A_ scores for acoustic CR neuromodulation therapy (black bar baseline, white bar after treatment) for two treatment phases. The first treatment phase (a) used only a CR neuromodulation device (T30 CR device with custom earphones only). The second treatment phase (b) used the same neuromodulation device (T30 CR) but combined with a streamer (Phonak ComPilot) wirelessly connected to hearing aids (Phonak Audeo V50).

In the first treatment phase (June 2012–November 2012) the measured tinnitus pitch fluctuated substantially with a mean pitch of 8040 Hz and standard deviation of 1400 Hz. During this treatment phase, tinnitus pitch matching was performed using a bracketing method, similar to the well-known Vernon-method [36]. Namely, at the six consecutive visits 7035 Hz, 9200 Hz, 6500 Hz, 9000 Hz, 6500 Hz, and 10000 Hz were used for the programming of the acoustic CR neuromodulation pattern. During the first phase of treatment the patient reported that his tinnitus perception had started to become more atonal in nature and reported that he was gradually becoming less confident regarding the accuracy of his pitch matching ability (i.e., therefore he exhibited a lack of confidence about the reliability of the result and a marked decrease in salience of the tonal aspect). The baseline initial THI score was 28 points, the VAS_L_ score was 80 points, and the VAS_A_ score was 70 points. During each of five follow-up visits the therapy signal was readjusted with respect to the measured tinnitus frequency. After five months of therapy the THI (44 points), VAS_L_ (90 points), and VAS_A_ (80 points) scores worsened by 57%, 13%, and 14%, respectively, Figure 3(a).

Another follow-up visit in February 2013 showed no change in tinnitus symptoms and therapy was stopped. In July 2015 hearing aids were fitted (Oticon Zest BTE digital instruments, Oticon A/S, Smørum, Denmark). The hearing aid was set up using a recent audiogram applying a national acoustic laboratory nonlinear prescription formula (NAL NL1) [37]. The patient had used hearing aids for the correction of his symmetrical presbycusis. No change in tinnitus symptoms was reported after wearing the hearing aids with no acoustic CR neuromodulation for 5 months (July 2015 to November 2015).

### 2.4. Second Phase of Treatment with Acoustic CR Neuromodulation

For the second treatment phase, the T30 CR device was connected via direct cable to a hearing aid streamer (Phonak ComPilot, Sonova AG, Stäfa, Switzerland) that wirelessly transmitted the therapy signals to hearing aids (Phonak Audeo V50, Sonova AG, Stäfa, Switzerland). The streamer sends an external audio signal (e.g., from an mp3 player, telephone, TV, or stereo system) through an external sound input to hearing aids that provide higher amplification customized to the patients hearing loss. The hearing aids (Phonak Audeo V50) were fitted with the same NAL NL1 prescription formula as used for the initial hearing aid (Oticon hearing aid Zest BTE digital instruments, Oticon A/S, Smørum, Denmark). This was done in order to ensure comparative amplification with the patient's original Oticon hearing aids used during phase one of treatment. Because the hearing loss was greater for high frequencies, in particular for frequencies above 6 kHz, the patient was not able to hear the highest stimulation tone, resulting in use of only the first three CR tones. Normally, this could cause the exclusion of the patient from treatment but was accepted for this case report. The tinnitus pitch was quite stable during the second treatment phase with a lower mean pitch of 6260 Hz and a much smaller standard deviation of 150 Hz. During this treatment phase, a newly developed automated and patented pitch matching method was utilized that integrated bracketing, similarity grading, a two-alternative forced-choice (2AFC) method, and fine-tuning into a single protocol of four distinct stages shown to improve pitch matching accuracy [38]. Namely, at the three consecutive visits 6109 Hz, 6109 Hz, and 6421 Hz were used for the programming of the acoustic CR neuromodulation pattern. The baseline score for THI was 40 points, for VAS_L_ was 92 points, and for VAS_A_ was 92 points. During two follow-up visits the therapy signal was readjusted to the slightly varying tinnitus frequency. After three months of therapy a large improvement of tinnitus symptoms was observed; namely, the THI score was reduced by 70% to 12 points, the VAS_L_ score was reduced by 32% to 63 points, and the VAS_A_ score was reduced by 32% to 63 points, Figure 3(b). The improvements of tinnitus symptoms were further substantiated by the additional results from another tinnitus questionnaire (TQ). The baseline TQ of 52 points was reduced by 35% to 34 points after 3 months of acoustic CR therapy applied through a hearing aid.

## 3. Discussion

During application of acoustic CR neuromodulation via a hearing aid a significant reduction in tinnitus symptoms was observed as documented by substantial decreases in VAS_L_, VAS_A_, and TQ scores. The magnitude of the hearing loss and the variable pitch matching results may have contributed to the unsuccessful initial acoustic CR neuromodulation treatment attempt. The application of acoustic CR neuromodulation through the hearing aids was well accepted and no side effects were observed throughout the intervention.

At the time of writing the majority of hearing instrument manufacturers provide aids with sound therapy features that can be utilized for a number of different therapeutic purposes and may serve to reduce the distress associated with tinnitus perception. Examples of these features include sound signals that are intended to be conducive to relaxation [39]; alternatively, others can be applied in order to provide some form of sound enrichment or masking. A low number of small-scale studies have reported that these systems can be useful for the management of tinnitus, with the supplementation of appropriate counseling [33].

Results obtained during this case report are encouraging from several clinical and technical standpoints. First, this approach is also applicable for tinnitus patients with sufficient hearing loss to warrant the use of hearing aids. Second, this approach allows simultaneously tinnitus therapy and hearing aid benefits. Third, this approach allows improved acoustic CR neuromodulation therapy signal control because of the inherent further amplification offered by the hearing aids used to treat the existing hearing loss. Fourth, this approach likely will result in higher compliance with the therapy regime because the primary device already is being worn. Fifth, the continued use of the hearing aid beyond acoustic CR neuromodulation therapy might help to stabilize the obtained therapeutic effects. Finally, this approach will allow easy implementation of additional CR therapy should the tinnitus return or worsen in the future.

There are some limitations with this approach. First, current hearing aids have a restricted frequency range that will limit the delivery of high frequency stimulation tones with high enough levels in cases of high frequency tinnitus. Second, the available data did not fully explain why the patient did not respond to CR therapy without hearing aid support in the first phase of the experiment. This might have occurred because of inadequate and imprecise pitch matching (see Section 2.3) or because of the substantial hearing loss close to the used stimulation tones. The slight worsening of the tinnitus symptoms might be linked to the patient's emotional reaction to not experiencing obvious improvement during therapy. Third, there may be some inconvenience related to the need for an optional “streamer” module. As an alternative, to cope with the latter disadvantage for further patients within this continued case series, devices are being developed [38] that allow the direct streaming of therapy signals to hearing aids wirelessly as is done for other sound therapies involving noise generators [32, 33].

## 4. Conclusion

In a single case, we were able to show that acoustic CR neuromodulation can be implemented effectively through contemporary hearing aids to allow the simultaneous benefits of hearing aid use and therapy delivery. This approach appeared to not negatively influence normal hearing aid functionality, increase the environments in which therapy can be delivered, and potentially increase likelihood of compliance with the tinnitus therapy regimen. Since only one case has been investigated so far, the results are clearly not yet conclusive. This case report encourages a more comprehensive investigation of the integration of acoustic CR therapy delivery with hearing aids including understanding the effects of nonlinear amplification, harmonic intermodulation, and temporal distortions inherent in contemporary hearing aids.
